# S06 Organisation of physical activity for people with acute or chronic diseases: the mechanisms, the barriers, and the possible solutions

**DOI:** 10.1093/eurpub/ckae114.221

**Published:** 2024-09-26

**Authors:** Juliet Harvey, Marie Crabbé, Jacqui Morris, Alexis Lion

**Affiliations:** University of Dundee; Artevelde University of Applied science; University of Dundee; Fédération Luxembourgeoise des Associations de Sport de Santé

## Abstract

**Purpose:**

Physical activity (PA) is a promising therapeutic tool to treat and manage several chronic diseases. However, few patients are engaged in regular PA or are sufficiently active.

This symposium investigated different mechanisms, barriers, and solutions which may explain the low PA engagement of patients with chronic diseases. Possible solutions will also be discussed.

**Project/policy description:**

The first presentation aimed to tackle the deconditioning during the acute phase of the treatment of chronic diseases in Scotland (UK) using a collaborative method. A Special Interest Group was formed and consisted in a collaborative hub where staff networked, supported each other, conducted projects, and shared their findings and challenges. It resulted in the development of resources, audit tools and training material.

The second presentation aimed to investigate the barriers explaining the lack of PA in cancer patients in Flanders (Belgium). Barriers experienced by patients and health and exercise professionals were identified and used to elaborate a tool allowing professionals to assist cancer patients more effectively in their exercise routines.

In third presentation aimed to developing and pilot testing a dyadic intervention to promote outdoor walking in people after stroke in Scotland (UK). The collected data illustrate the potential of dyadic interventions with this clinical population. The data highlight the need for careful matching of dyads, facilitation of dyadic working by practitioners who can support development of dyadic relationships; and community structures linked to healthcare pathways that support implementation.

The last presentation aimed to understand how outpatient PA for cardiac patients is organised in Luxembourg and its 3 surrounding countries. Organisation was different in the 4 countries. Guidelines on the organisation of the outpatient PA for cardiac patients are needed to harmonise the organisation with may help to increase the participation.

**Conclusions:**

Organisation of the PA offer for people with chronic diseases, which include a better promotion from healthcare professionals and more patient-centred programmes/initiatives, may increase the participation. These examples will open a discussion between the speakers and the audience attending the symposium. It will feed into the reflections of the Working Group on HEPA Promotion in healthcare settings.

